# Skin transcriptome reveals the periodic changes in genes underlying cashmere (ground hair) follicle transition in cashmere goats

**DOI:** 10.1186/s12864-020-06779-5

**Published:** 2020-06-05

**Authors:** Feng Yang, Zhihong Liu, Meng Zhao, Qing Mu, Tianyu Che, Yuchun Xie, Lina Ma, Lu Mi, Jinquan Li, Yanhong Zhao

**Affiliations:** College of Animal Science, Inner Mongolia Agricultural University, Hohhot, 010018 China

**Keywords:** Transcriptional group, Differentially expressed genes, Cashmere goat skin, Villus growth cycle, Keratin

## Abstract

**Background:**

Cashmere goats make an outstanding contribution to the livestock textile industry and their cashmere is famous for its slenderness and softness and has been extensively studied. However, there are few reports on the molecular regulatory mechanisms of the secondary hair follicle growth cycle in cashmere goats. In order to explore the regular transition through the follicle cycle and the role of key genes in this cycle, we used a transcriptome sequencing technique to sequence the skin of Inner Mongolian cashmere goats during different months. We analyzed the variation and difference in genes throughout the whole hair follicle cycle. We then verified the regulatory mechanism of the cashmere goat secondary hair follicle growth cycle using fluorescence quantitative PCR.

**Results:**

The growth cycle of cashmere hair could be divided into three distinct periods: a growth period (March–September), a regression period (September–December), and a resting period (December–March). The results of differential gene analyses showed that March was the most significant month. Cluster analysis of gene expression throughout the whole growth cycle further supported the key nodes of the three periods of cashmere growth, and the differential gene expression of keratin corresponding to the ground haircashmere growth cycle further supported the results from tissue slices. Quantitative fluorescence analysis showed that *KAP3–1, KRTAP 8–1,* and *KRTAP 24–1* genes had close positive correlation with the cashmere growth cycle, and their regulation was consistent with the growth cycle of cashmere.

**Conclusion:**

The growth cycle of cashmere cashmere could be divided into three distinct periods: a growth period (March–September), a regression period (September–December) and a resting period (December–March). March was considered to be the beginning of the cycle. KAP and KRTAP showed close positive correlation with the growth cycle of secondary hair follicle cashmere growth, and their regulation was consistent with the cashmere growth cycle. But hair follicle development-related genes are expressed earlier than cashmere growth, indicating that cycle regulation could alter the temporal growth of cashmere. This study laid a theoretical foundation for the study of the cashmere development cycle and provided evidence for key genes during transition through the cashmere cycle. Our study provides a theoretical basis for cashmere goat breeding.

## Background

Hair follicle growth in mammalian skin changes dynamically after birth and continues in a cyclical pattern. The hair growth cycle can be divided into three phases: telogen, anagen, and catagen [1–5], each of which is regulated by specific genetic patterns [6, 7]. Inner Mongolian cashmere goats have two distinctly different fibrous hair structures, with thick, coarse guard hairs forming the outer layer and fine, soft ground hairs forming the cashmere underneath. The cashmere comes from secondary hair follicle structures in the skin [8], and the coarse hair comes from primary hair follicles [9, 10]. Hair follicles, after shedding their old hair shafts, produce new hair shafts [11], thereby starting a new cycle of hair growth [12]. Keratin (KRT) and keratin-associated proteins (KRTAPs) are the main components of hair and affect its physiological properties. Hair follicle and hair shaft growth involve changes in the expression of genes encoding a KRT intermediate silk protein and a KRTAP [13–16]. Human hair follicles do not have a synchronized growth pattern, with each hair follicle being independent of others [17]. In contrast, the growth of cashmere goat hair exhibits periodic changes with annual changes in daylight [17, 18]. This periodic growth pattern depends on intrinsic molecular mechanisms and the external environment [19, 20].

With the progress of research technology, second-generation high-throughput sequencing technology can be used to screen for differentially expressed genes [21], allowing research across a broad spectrum of gene expression. RNA-Seq technology is a high-throughput sequencing technology that can be used to discover low abundance transcripts and new transcripts and to identify differential expression of transcripts among different samples [22–24].

In this study, second-generation high-throughput sequencing technology was used for transcriptomic sequencing of skin samples from different stages of hair growth. The aim of this study was to investigate the correlation between differentially expressed genes and the regulation of hair cycle transitions at different stages of hair growth. The biological functions of differentially expressed genes at different stages of hair growth play an important role in elucidating the regulatory mechanisms of hair growth, laying a theoretical foundation for its study.

## Results

### Morphological analysis of hair cycle changes in goat skin

**Fig. 1:**
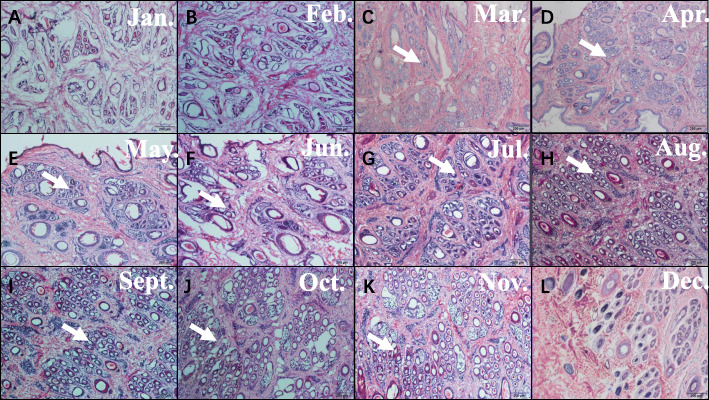
Morphological study of skin tissue from cashmere goats over 1–12 months. Hair follicles began to be produced in March, cashmeres began to be produce in June, Cashmere visible outside the epidermis in July, and hair follicle structure began to decline in December

We made a histological examination of skin tissue from cashmere goats, as follows. Results showed (Fig. 1), that the number of secondary hair follicles in cashmere goats decreased gradually from December to March (Fig. 1l, A, B, C). The lowest value was reached in March (Fig. 1c), and the statistical values of each trait were also lower. The division and extension of hair follicles to the dermis began in April (Fig. 1d), while the number of secondary hair follicles also began to increase at the same time. A velvet-like appearance to the goat’s coats was observed in the month of July (Fig. 1g). Most cashmere grew from follicles in the skin between August and September (Fig. 1h, i). At the same time, the number of secondary follicles reached its highest level, with this period being considered as the peak period of cashmere growth. In October, hair follicle bulb cells began to enlarge, gradually aged and died, and the dermal papillae began to atrophy. The numbers of secondary hair follicles gradually decreased (Fig. 1j). In December, the hair follicle roots rose to the sebaceous glands, and the secondary follicle numbers reached their lowest level (Fig. 1l), This state was maintained until February of the following year. Fro this information, we made the initial inference that the cycle of secondary hair follicles in cashmere goats can be divided into a growth period from March to September, a resting period from September to December, and a regression period from December to March. Generally speaking, we divided the cashmere hair cycle into three periods by observing skin tissue morphology, but the key points of each time period could not be determined; this needs further study.

### Differential gene expression analysis

**Fig. 2:**
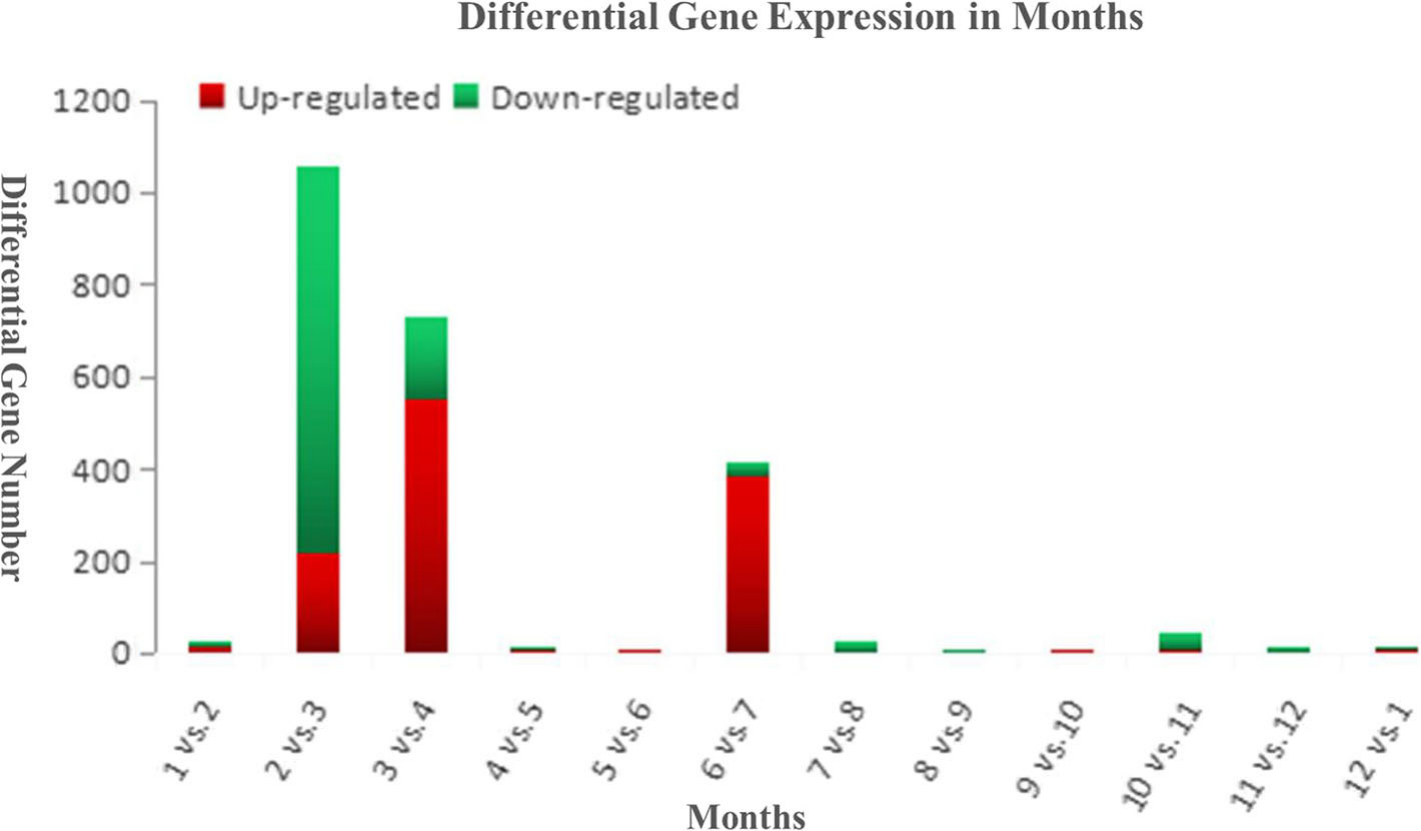
Histogram of differentially expressed gene statistics between neighboring months. Significant changes in gene expression occurred in March. In July, cashmeres began to grow above the skin surface, and gene expression changed significantly. The results of gene expression tended to be stable at other times, and negative regulation was dominant

We first screened and analyzed data quality (Table 1) and data length distribution (Table 2). Then we compared the skin transcriptome data of cashmere goats in 12 months with neighboring months (Fig. 2). It was found that the number of differentially expressed genes was the greatest between February and March. The total number of differential genes was 1059, of which 219 were up-regulated and 840 were down regulated. In March and April, the number of differentially expressed genes was 731, of which 550 were up-regulated and 181 were down regulated. In June and July, there were 418 differentially expressed genes, of which 388 were up-regulated and 30 were down regulated. These results showed that the expression of genes was initially up-regulated or down-regulated during the initiation of secondary hair follicle growth. Along with advancement in hair follicle initiation, the number of down-regulated genes began to decrease, and the number of up-regulated genes continued to increase. After completion of the initiation process, the gene changes tended to be stable. The results further showed that hair follicle development was initiated by a combination of up-regulation and down-regulation of genes in the early stage of initiation, and that gene expression returned to normal levels after initiation. Comparing the data between June and July, we found that there was another significant change in gene expression during cashmere outgrowth. We believe that this change promoted cashmere to emerge from the skin surface, but the existence of other roles remains to be studied. From August to February of the following year, secondary hair follicle gene expression changed significantly from quiescence to degeneration. These results further showed that the initiation of secondary hair follicles in cashmere goats began in March. Finally, it is worth mentioning that the number of differentially expressed genes increased first and then decreased from February to March, and then to April, thus emphasizing that the secondary follicle cycle starts in March.

**Table 1:** Primer sequences and fragment size of Cashmere goat *KRTAP3–1, KRTAP8–1, KRTAP24–1* gene and β-actin

Gene Name	Sequence of primer	Products size
*KRTAP3–1*	F: CACACGACATCAGCCTCCT R: GGTGGGAAGAGTTGAGCAGA	108 bp
*KRTAP8–1*	F: TTCTCCAGCACCGTCTTCC R: TAGCCATAGCCGAAGCCATA	122 bp
*KRTAP24–1*	F: CTC TTT GCT CCA GCG ATG TAA R:AGG GCA CAG ACG AGT TTG A	183 bp
*β-actin*	F: GGCAGGTCATCACCATCGG R: CGTGTTGGCGTAGAGGTCTTT	158 bp

**Table 2:** The result of the high quality raw data

Sample name	Data quantity (Mb)	Reads number	Base number
January	1943	19,823,380	1,942,915,104
February	1951	19,853,076	1,951,357,594
March	2104	21,419,793	2,103,697,829
April	1878	19,309,914	1,878,190,553
May	1986	20,231,833	1,986,356,467
June	1961	19,961,875	1,960,645,956
July	2479	25,514,634	2,479,016,770
August	1877	19,105,505	1,877,107,615
September	2140	21,779,658	2,139,505,645
October	2112	21,506,614	2,112,499,444
November	2301	23,688,064	2,300,596,485
December	2040	20,939,374	2,039,994,952

### Classification of gene function annotation

**Fig. 3:**
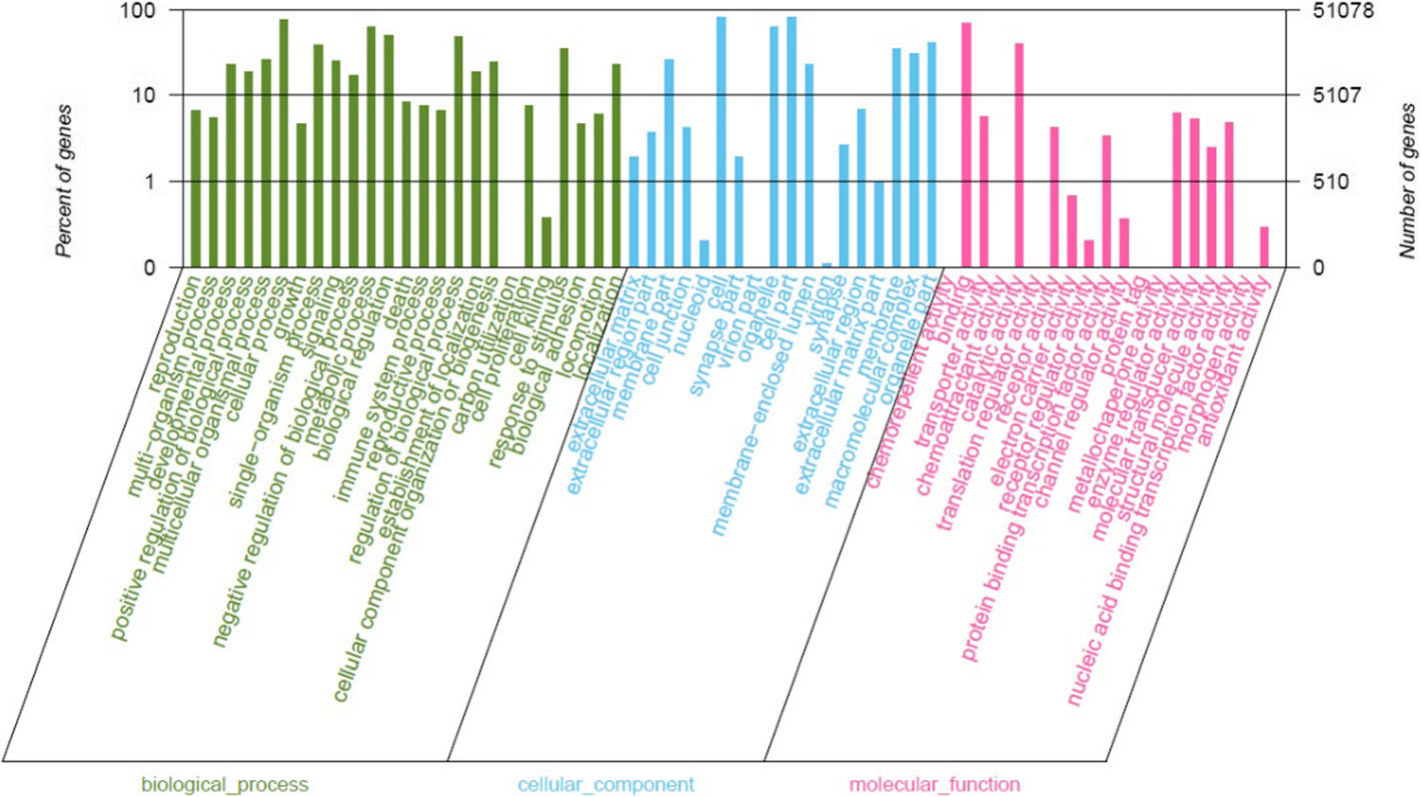
Gene ontology annotation

According to GO classification statistics (Fig. 3), skin expression genes can be divided into three main categories: biological functions, cell components, and molecular functions. In this study, 51,078 transcripts were noted using GO annotation. Among them, in biological function, the most annotated transcript was cellular process. In cellular component, most of the transcripts were transcribed to cell and cell part. In molecular function, most of the transcripts were transcribed to binding. It is speculated that during the hair follicle cycle, the changes of gene expression led to changes in the number and state of cells in hair follicles, which further led to the occurrence or shedding of secondary hair follicle.

### Group clustering analysis of natural periodic samples

**Fig. 4:**
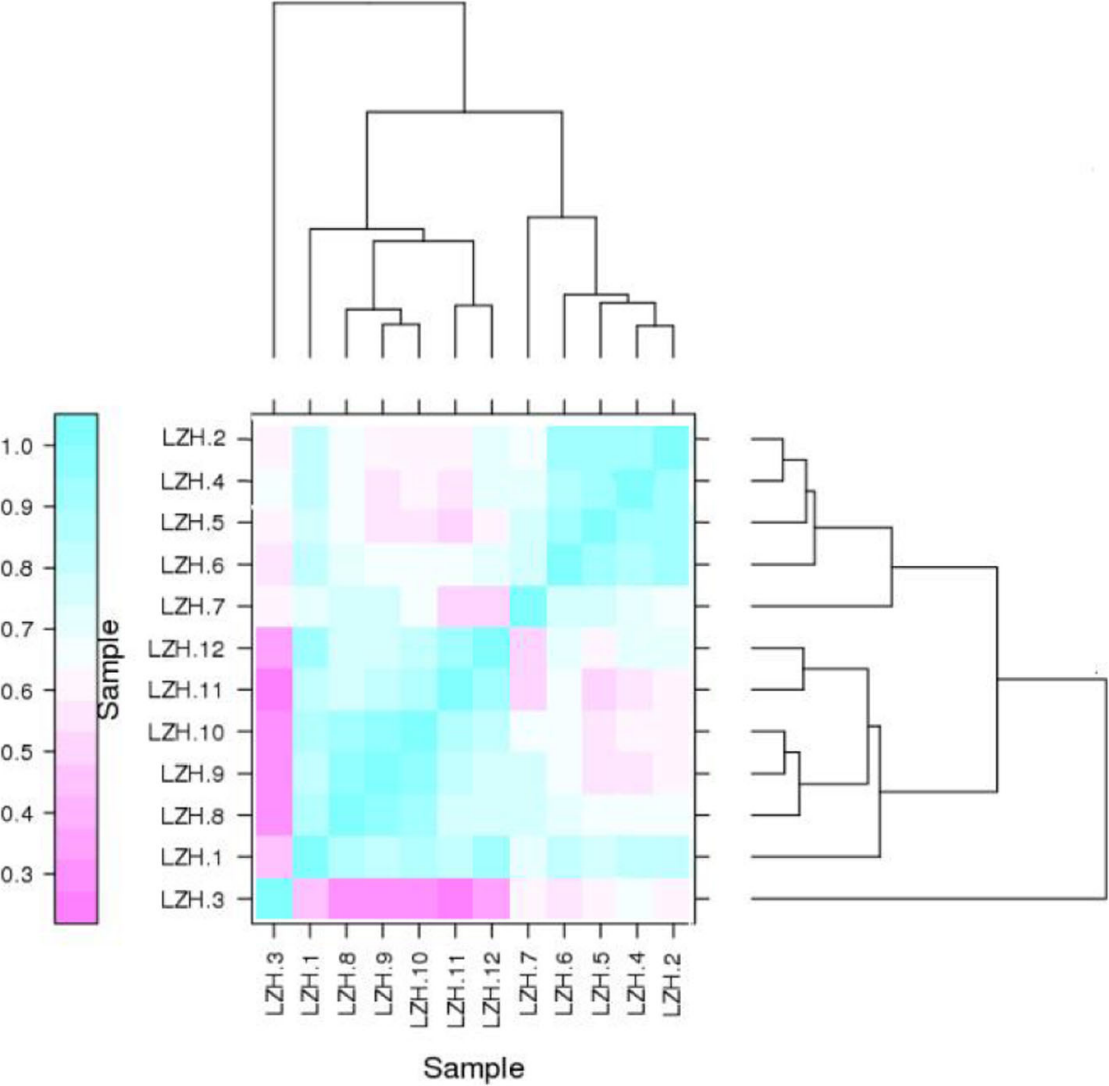
Cluster diagram of the growth cycle of cashmere. Clustering results divide 12 cycle samples into three main categories

In order to further explore the rule of gene expression, we calculated the correlation coefficient and cluster analysis of all gene expression levels in the 12-month natural cycle (Fig. 4). The results showed that clustering information could be divided into three categories. The sample LZH3 was isolated because of the great changes in gene expression of the follicle promoter. Samples LZH2–LZH7 were considered to be the initiation process of hair follicle growth. Samples LZH8–LZH12 were clustered together because they were thought to control the transition of secondary hair follicles from vigorous growth to recession. Gene expression remained relatively unchanged from December to March, so the clustered sites had reached the end of degeneration and before growth; this was considered to be the resting period of hair follicle development. Combined with previous studies in this manuscript, we found that there were several critical periods in the division of the secondary hair follicle cycle. March is considered the key point for initiation of the hair follicle cycle; September is the key period for vigorous growth and the beginning of recession; December is the key point for the end of hair follicle recession and the beginning of the rest period, These three critical periods were determined by key signals in hair follicle and cashmere growth.

### Extraction and analysis of target gene expression information

**Fig. 5:**
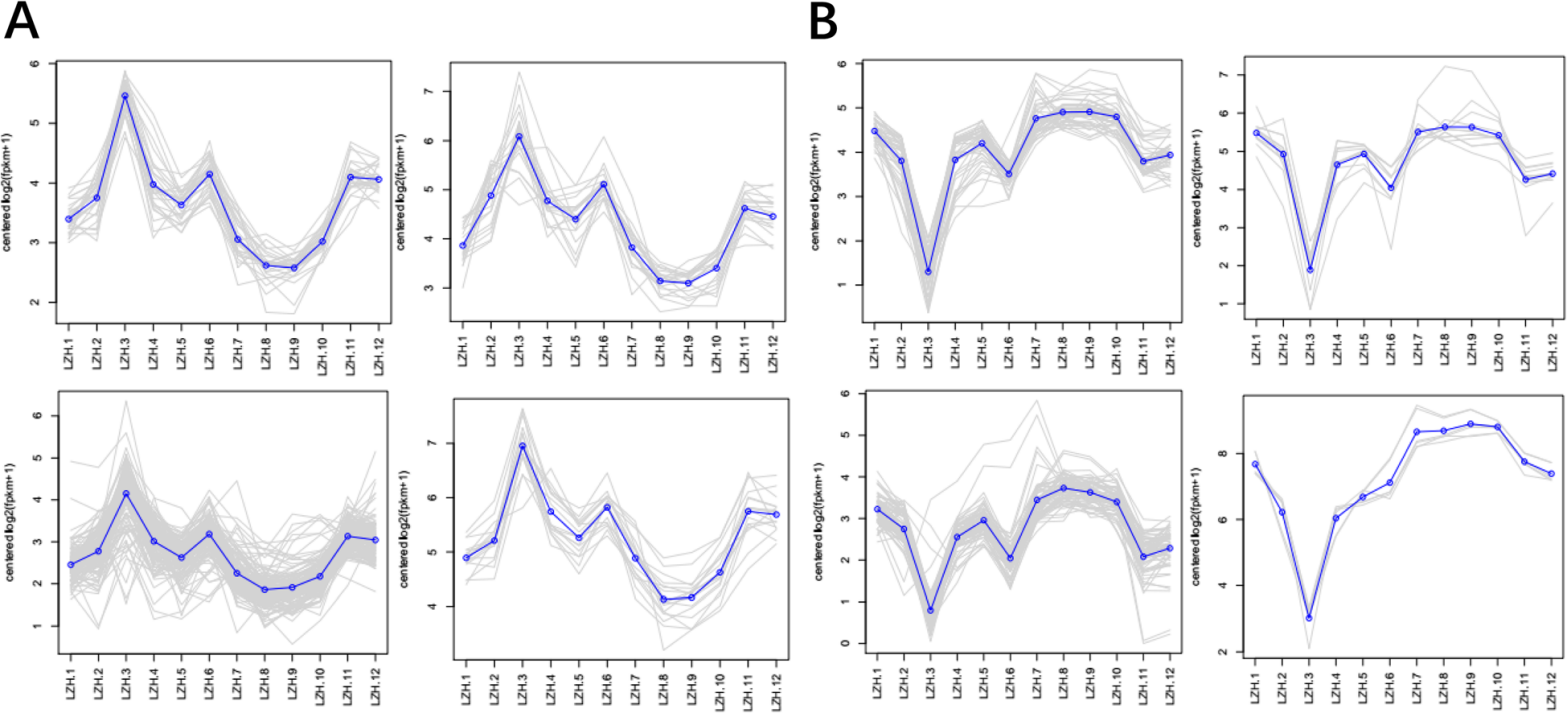
Clustered expression patterns

In order to explore the expression patterns of genes that play key roles in the cycle, we extracted expression information of all target genes for 1–12 months, and then clustered the expression patterns by analysis and exclusion. Gene expression patterns of several pathways related to the cashmere cycle were obtained (Fig. 5a). Results showed that gene expression patterns related to the cashmere cycle were consistent with our analysis of differential gene expression. The results further supported the previous finding that the hair follicle cycle was initiated in March, entered the regression stage in September, and entered the end stage in December. However, transition through the hair follicle cycle cannot be visualized through skin histology, because the cycle initiation precedes cashmere growth, and there is a causal relationship between them. The period of cashmere growth was observed in tissue sections, and there was a direct relationship between cashmere growth and the expression of keratin. Therefore, in order to further verify the cashmere growth cycle, we also clustered the expression patterns of keratin and keratin-related genes (Fig. 5b). The results showed that the expression of keratin was consistent with the results of tissue sections, which further supported our findings that the hair follicle cycle first started (degenerated or rested), and then cascaded, leading to changes in the expression of the keratin gene, thus promoting the occurrence of cashmere (growth or degeneration). The gene expression characteristics can be divided into two types. First, the expression of apoptosis-related genes showed a downward trend from the resting stage to the early growth stage (Fig. 5b, LZH3). Secondly, the expression of genes increased which related to hair follicle development (Fig. 5a, LZH3) or decreased after the development of hair follicles during the growth period (Fig. 5b, LZH6), and the expression of genes related to controlling cashmere growth increased (Fig. 5a, LZH6). In general, development of hairfollicles and cashmere growth showed a wave-like expression. In order to further study the relationship between the keratin gene and cycle, we selected the gene with the highest expression as a case for further study.

### QPCR analysis of key genes

**Fig. 6:**
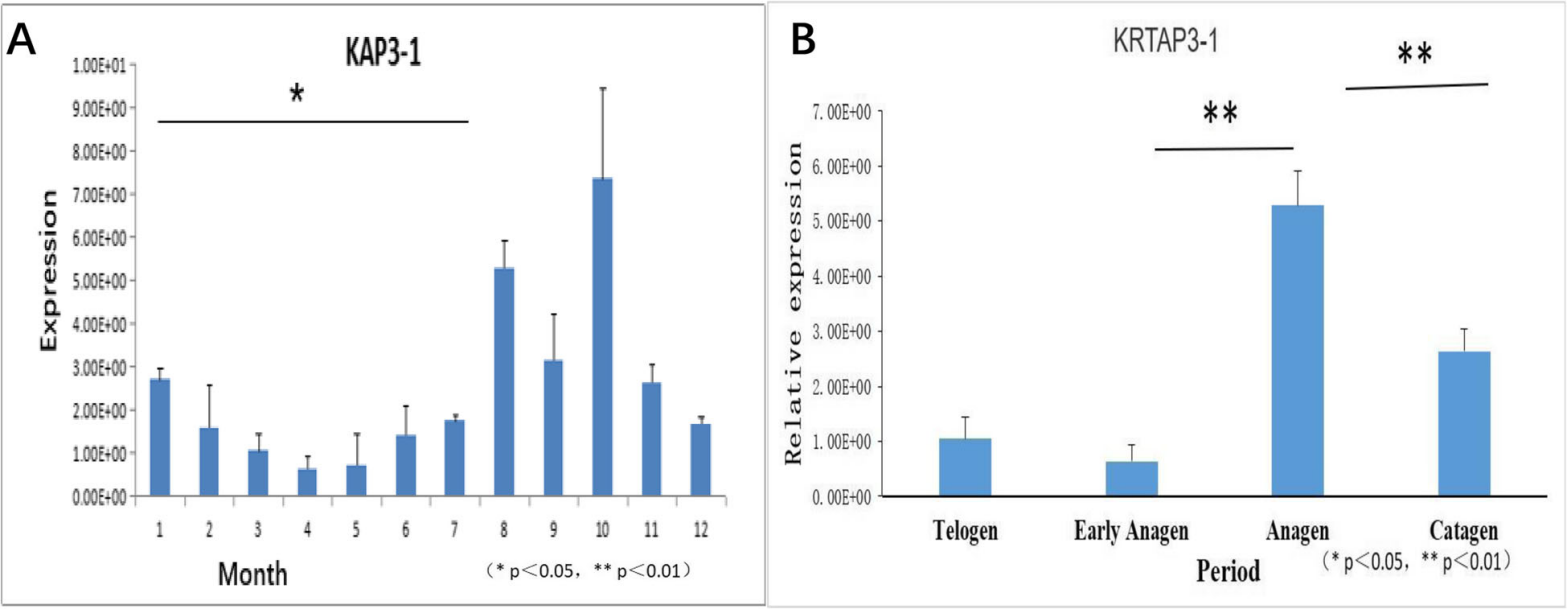
Differential expression of *KRTAP 3–1* in different months and periods

**Fig. 7:**
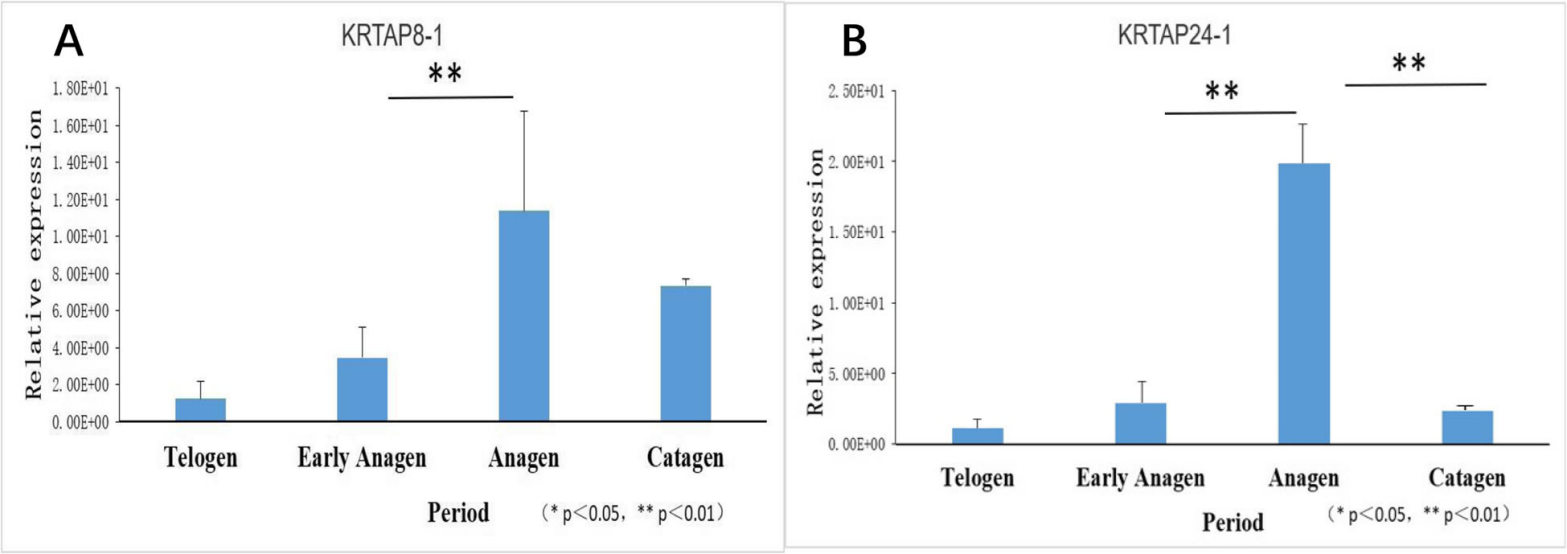
Differential expression of *KRTAP 8–1* and *KRTAP 24–1* in different periods

Among keratin and keratin associated proteins, keratin associated protein 3–1 ranked first in cluster 11 with an expression level of 80,824 at the growth stage and 23,856 at rest stage. The expression of keratin associated protein 3–1 in cashmere for 12 months was confirmed by quantitative PCR (Fig. 6a). The results showed that *KAP 3–1* was expressed in the skin at different stages of the year, its expression was significantly different (*P* < 0.05) and fluctuated periodically in a 12 month period. Expression levels in the 3 months of August, September, and October were significantly higher than that in other months (P < 0.05). Combined with previous studies, the expression quantity was verified by investigating different periods (Fig. 6b). It was found that expression of the *KAP3–1* gene in the growth phase was significantly higher than that in either the rest or regression phases. Subsequently, to verify the stability of gene expression, we examined the expression of two other genes, *KAP 8–1* and *KAP 24–1,* in cashmere goat skin at several stages using fluorescence quantitative analysis (Fig. 7). The relative expression of *KRTAP 8–1* (Fig. 7a) and *KRTAP 24–1* (Fig. 7b) genes in Inner Mongolian cashmere goat skin showed periodic variation, which was consistent with the hair follicle development cycle. This indicated that *KRTAP 8–1* and *KRTAP 24–1* genes played a positive role in controlling cashmere wool growth and were closely related to the regulation of cashmere growth and cycle transformation.

## Discussion

Under natural conditions, animal hair displays a regular growth pattern and, following birth, the hair follicles are constantly changing [25, 26]. The follicles undergo self-renewal and periodic growth, which can be divided into the three phases of anagen, catagen, and telogen [27–29]. Therefore, it is important to study the changes in cashmere goat hair follicles and the differential expression of their regulatory genes to improve the production and thus the economic value of cashmere goats.

Secondary hair follicle development of cashmere goats is a cyclical process [30]. In this study, the histological slices of cashmere goat secondary follicles showed that skin thickness, the length and depth of primary follicles, and the width, density and activity of primary follicles did not change significantly from January to March; the statistical value of each trait was low. From April onwards, cell division in the roots of hair follicles accelerated and extended to the dermis, and morphological data began to increase until July, when the cashmeres started to protrude above the skin surface. In August and September, most of the cashmeres continued to lengthen above the skin. At this time, most of the statistical values of follicle characteristics reached an annual maximum, which indicated the peak period of cashmere growth. From October, hair follicle globular cells began to degenerate and die, and hair papilla cells began to atrophy. At this time, the statistical values of hair follicle morphological data began to decline. In December, the root of hair follicles rose to the vicinity of the sebaceous gland, and the statistical values of hair follicles reached their lowest level for the whole year, and remained there until February of the following year. From this, we inferred that the growth period of cashmere goat hair follicles is from March to September, the regression period is from September to December, and the rest period is from December to March. These results are consistent with previous preliminary studies [20, 31–33].

Inner Mongolia cashmere goat, as a local breed, is very different from the conventional goat in the type of hair. There are a lot of genes starting with LOC in the mapping of goat reference genome. Through NCBI database, we found that many of these genes are related to hair follicle development. In order to further study the differences between the existing goat reference genome and de novo assembled scripts, we compared the two methods, and found that the number of genes obtained by de novo assembled scripts was much more than that obtained by mapping of goat reference genome. After that, we compared mapping goat reference genome with GFT and Without GFT, and found that 36,293 total genes and 40,556 total genes were obtained respectively. And for de novo assembled scripts, velvet and Trinity methods are used respectively. and 323,630 total scripts and 511,110 total scripts were obtained. From this result, for Inner Mongolia cashmere goats, the result of de novo assembled scripts is better than that of mapping existing genome. So we de novo assembled scripts here.

Through transcriptome sequencing technology, we can identify the changing roles of skin expression genes in cashmere goats at different stages and the levels of expression at different stages [21, 34, 35]. Transcriptome sequencing can guide us to study the direction of the hair follicle cycle using gene expression. However, we observed the phenotypic characteristics of hair follicles using tissue sections, and initially explored the expression regularity of the hair follicle cycle and the differences of hair follicle characteristics in different periods. Subsequently, transcriptome sequencing was used to detect gene expression and expression pattern, and the expression levels and expression patterns were used to verify the results of tissue sections. Finally, the results of gene expression were verified by fluorescent RT-qPCR. This reverse validation was used to ensure the accuracy of phenotypic traits and gene expression patterns, more accurately locating and studying those genes related to the hair follicle growth cycle. The results of this study showed that the number of secondary hair follicles began to increase from March. The results of transcriptome sequencing showed that there were significant changes in gene expression in March compared with February: gene expression increased, and the number of differentially expressed genes increased. These two results verified each other and suggested that March was the start of the secondary hair follicle cycle. In September, the cashmere grew to its peak and then began to decrease, and the number of secondary follicles also began to decrease. Meanwhile, gene expression was first down-regulated and then up-regulated in the 3 months from August to October; gene changes were obvious in September, which indicated this was the end of the cashmere growth cycle and the beginning of degeneration. The number of hair follicles in the degenerative period lasted until December, and then the number of secondary hair follicles remained unchanged from December to early March of the following year, that is, the period of the end of the cashmere growth cycle. At present, there are many comparative studies examining differential gene expression and differentially expressed genes (DEGs) in cashmere goats, mainly focusing on the role of differential genes produced by DEGs in gene expression, such as the role of the *HOX13* gene in cashmere growth, but few studies examining cycle division have been reported. In this paper, the occurrence and decline of cashmere hair were accurately divided according to its development, which was helpful to study the hair follicle cycle in cashmere research.

Compared with a marginal analysis at each time point, Hidden Markov models demonstrate a substantial increase in sensitivity, with little increase in the false discovery rate. So we tried to use Hidden Markov Models to analyzed the differential genes, but in this paper, our aim is to propose the growth cycle of cashmere in Inner Mongolia cashmere. It is well known that the growth of cashmere is regulated by the development of hair follicles and the genes and signaling pathways that change in the early stage. The cycle of each cashmere follicle occurs. First, the expression of genes in hair follicles changes, stimulating hair follicles, and then further genetic changes, leading to the growth and decline of villi. That is to say, the time when the gene changes is earlier than the time when the villi on the surface of the body changes. Hidden Markov Models analysis can be the most perfect analysis of regulatory gene expression, but in time earlier than the occurrence of villus, so it can not match the tissue slice of villus growth in our number 1 very well. Therefore. We have to adopt the sub-optimal comparative analysis scheme. Although this can not better explain the variation of differential genes, it is more obvious to find the critical time point of hair follicles cycle change.

Differential expression analysis of genes is very important for later data analysis and accurate results. As shown in Fig. 2, we considered using Hidden Markov models [36, 37] to analyze differential genes. But in this paper, our aim was to propose a growth cycle for cashmere in Inner Mongolian Cashmere goats. It is well known that cashmere growth is affected by the development and early changes of hair follicles; the regulation of genes and signaling pathways changes only later. In each cashmere follicle cycle, gene expression in the follicle changes first, stimulating the occurrence of follicles, and then further genetic changes, leading to the growth and decline of cashmeres. That is to say, the timing of gene change is earlier than the timing of change in observable cashmere. Hidden Markov Model analysis is the most perfect way to analyze the expression of regulatory genes,. Therefore. We had to adopt a sub-optimal comparative analysis scheme. Although this could not give a better explanation of the variation of differential genes, it was a more obvious way to determine the critical time points of cashmere cycle change.

KRT and KRTAPs together make up nearly 90% of the cashmere yield [38–40], demonstrating indirectly that the composition and interactions of these proteins play an important role in cashmere quality [41–43]. *KRT* and *KRTAP* gene expression directly affect the fineness and density of cashmere as well as other characteristics [44]. Most of the genes identified by transcript sequencing of Inner Mongolian cashmere goats were members of the *KRT* and *KRTAP* gene families, which indicated that the expression of KRTAPs directly affected the growth of hair follicles and cashmere-related traits.

Thus, the cycle of hair follicle growth is correlated with a variation in gene expression, and the complex regulates the cycle of hair follicle growth [45]. In addition, there was a sharp fluctuation in the expression of genes during telogen and anagen. However, genetic variation in the growth of cashmere stops growth relatively slowly, making the process gradual. Therefore, the study of genes related to the initiation of cashmere growth have potential value in the discovery of genes that affect cashmere growth, such as those that regulate the cashmere cycle.

## Conclusion

The growth cycle of cashmere hair could be divided into three distinct periods: a growth period (March–September), a regression period (September–December) and a resting period (December–March). March was considered to be the beginning of the cycle. KAP and KRTAP had a close positive correlation with the cashmere growth cycle, and their regulation was consistent with growth. However, their expression time was different, indicating that regulation of hair follicle made the change of cashmere growth. This study laid a theoretical foundation for further research into the cashmere transitional cycle and provided evidence for key genes in cashmere development. It offers a theoretical basis for cashmere goat breeding.

## Methods

### Animals

In this experiment, Inner Mongolian cashmere goats were selected from a grazing environment of Inner Mongolian Arbas white cashmere goats at Jin Lai Breeding Farm. All animal experiments were performed in accordance with the “Guidelines for Experimental Animals” of the Ministry of Science and Technology (Beijing, China). All surgery was performed according to recommendations proposed by the European Commission (1997) and was approved by the experimental animal ethics committee of the Inner Mongolia Agricultural University. Samples for each month are three adult cashmere goats at a time under the conditions of similar growth, the same age and the same feeding conditions, and then mix the RNA of samples. The skin samples of 36 cashmere goats were collected from the same family. And they have the same father. The samples were collected from the middle part of the scapula at 10–15 cm in the Department of Surgery, and the experimental animals treated the skin with drugs after sampling, which did not affect the normal growth of the animals, and were not sacrificed. Directly after collection, the 3-cm-diameter skin samples were flushed with PBS and rapidly frozen in liquid nitrogen. Then, the samples were transferred to the laboratory in a liquid nitrogen tank and stored in a − 80 °C freezer.

### Total RNA extraction from skin

Total RNA was extracted from the skin of three cashmere goats using an RNAiso Plus Kit (TRIzol method). Total RNA was tested for purity and integrity using a sterile UV-vis spectrophotometer and an Agilent 2100 bioanalyzer, respectively, and then the three RNA samples were mixed. Total RNA was stored in a freezer at − 80 °C.

### Construction of sequencing library

cDNA library construction for transcriptome sequencing was performed according to the operating instructions for the Illumina TruSeqTM RNA Sample Preparation Kit. The total RNA from the three cashmere goats was pooled by mixing equal amounts of each. The mRNA was purified using oligo-dT magnetic beads and then fragmented to 100–400-bp mRNA. Double-stranded cDNA was synthesized by using fragmented mRNA as a template, exonucleases, and polymerases. The ends of the double-stranded cDNA fragments were blunted, and the double-stranded cDNAs were phosphorylated to ligate the sequencing adapters and poly (A) tail, and the sizes of the cDNA recovered were confirmed to be 200–300 bp by using a Bio-Rad Certified Low-Range Ultra Agarose Kit. PCR amplification of cDNA was performed to obtain a sequencing library, and library quality control was conducted using a TBS-380 instrument.

### Transcriptome sequencing

Paired-end sequencing of the cDNA was conducted by using an Illumina HiSeqTM 2000 sequencing platform. A 2 × 100 bp sequencing test was performed, and samples were sequenced by Shanghai Meiji Biopharmaceutical Co., Ltd.

### Quality and stitching of sequencing data and annotation of stitching results

Overlapping and low-quality sequencing data were removed. Trinity software was used to assemble the data into transcripts for transcriptional annotation and calculation of expression levels. The annotated sequences were aligned with the GO database using Blast2GO software, and annotated sequences were classified according to biological processes, cellular components, and molecular functions. The expression levels were calculated using FPKM (fragments per kb of transcript per million mapped reads) [24], and finally, significant difference analysis was performed on the expression levels of all genes/transcripts in each group of samples. RSEM and R software were used to identify all the differentially expressed genes/transcripts, and R software was used to calculate the relative expression levels of the genes.

### Hematoxylin and eosin staining of skin samples over twelve months

Fresh skin samples were taken from Inner Mongolian cashmere goats over 1 year and fixed in 4% paraformaldehyde for 24 h, which was followed by dehydration in a 30% sucrose solution for approximately 24 h. The samples were embedded in OCT, frozen, and sectioned on a cryostat. The 6-μm-thick sliced tissue samples were stained with hematoxylin for 1 minute and rinsed with water. After washing, each sample was placed for 3 s in ethanol with 1% hydrochloric acid and then in 1% alkaline ethanol. Eosin staining was performed when the alcohol gradient was dehydrated to 95%, and dehydration was continued after staining. Finally, the samples were treated with xylene and neutral gum and observed under a microscope.

### Quantitative reverse transcription PCR

The cDNA obtained above was used for quantitative reverse transcription PCR (q-PCR) analysis. The gene-specific primers used for q-PCR were designed with Primer 3.0 software and synthesized by Sangon Biotech Co., Ltd. (Shanghai, China). The primer sequences and fragment sizes are listed in Table 3. The q-PCR was performed using a PrimeScript RT Reagent Kit (TaKaRa, Beijing) in a 20-μL reaction volume with 10 μL of 2× SYBR Premix Ex Taq II (TaKaRa), 2 μL of cDNA, and 0.5 μL of each primer. The reaction was assessed on a Bio-Rad IQ5 multicolor real-time PCR detection system (Hercules, CA, USA). The β-actin ware was used as a reference. The qRT-PCR analysis was performed using the 2 − ΔΔCT method, and statistical analysis was performed using SPSS software (version 17.0). Values are represented as mean ± standard deviation. A significance level of 0.05 was used.

**Table 3:** Data of sequence length distribution

Isogene length	Isogene number	Percentage(%)
1–400	7737	7.31
401–600	19,281	18.21
601–800	11,147	10.53
801–1000	7796	7.36
1001–1200	6054	5.72
1201–1400	5041	4.76
1401–1600	4545	4.29
1601–1800	4165	3.93
1801–2000	3755	3.54
2001–2200	3336	3.15
2201–2400	3076	2.91
2401–2600	2842	2.68
2601–2800	2624	2.48
2801–3000	2342	2.21
3001–3200	2106	2.00
3201–3400	1881	1.78
3401–3600	1802	1.70
3601–3800	1642	1.55
3801–4000	1430	1.35
4001–4200	1378	1.30
4201–4400	1182	1.12
4401–4600	1047	1.00
ALL	105,854	100

## Data Availability

All data are saved in NCBI SRA (sequence readarchive) and SRA accession numbers is: SRP145408. The DNA sequence of the *Capra hircus KRTAP3–1, KRTAP8–1, KRTAP24–1* genes, obtained from NCBI/GENE (NCBI Reference Sequence is NC_030826.1, NC_000021.9, NC_000021.9), was compared against that of NCBI/BLAST.

## Abbreviations

KRT: Keratin
KAP: Keratin-associated protein
KRTAP: Keratin-associated protein
qRT-PCR: Quantitative real-time PCR
GO: Gene ontology
KEGG: Kyoto Encyclopedia of Genes and Genomes
PBS: Phosphate buffer saline
FPKM: Fragments per kb of transcript per million mapped reads
